# Rethinking youth emergency mental healthcare in the UK: insights from Australian service models

**DOI:** 10.1192/bjb.2025.10130

**Published:** 2025-10

**Authors:** Daniel Romeu

**Affiliations:** Faculty of Medicine and Health, University of Leeds, Leeds, UK

**Keywords:** Child and adolescent psychiatry, emergency care, mental health services, programme theory, scientific realism

## Abstract

Emergency mental healthcare for young people in the UK has been described as fragmented, risk-driven and under-resourced. Drawing on insights from Australian service models, this editorial explores how timely, integrated and relational care can improve outcomes and reduce harm. Key innovations, including early intervention hubs, assertive aftercare, outreach models and telehealth, are examined through a realist lens to explain how and why they work. Recommendations are offered for rethinking the strategy and provision of youth crisis care in the UK, centred on developmental need, relational continuity and a departure from risk assessment tools that lack an evidence base.

Providing effective emergency mental healthcare presents a critical challenge across global health systems, particularly for young people. In the UK, increasing numbers of young people present to emergency services in acute distress and care is often fragmented, reactive and shaped by institutional barriers instead of therapeutic need.^1^ Clinical encounters that should offer safety and support to young people and their families instead often cause iatrogenic harm and risk entrenching shame and disengagement. There is a growing call for alternative models that offer integrated, proactive and youth-centred care. Although not without its challenges, the Australian healthcare system has made significant strides towards improved accessibility, acceptability and efficacy of services. This editorial draws on field observations and discussions with patients and service providers during a research visit to three cities in Australia, alongside emerging programme theories explaining how and why emergency services help (or hinder) young people. Recommendations are offered for implementation in the UK context.

## Structural differences: age, access and risk

The contrasts between UK and Australian systems are stark. UK mental health services retain a child/adult binary, with support often abruptly ceasing on a young person’s 18th birthday. This is despite evidence that 18- to 25-year-olds face the widest gaps between mental health need and provision.^2^ By contrast, many Australian services now adopt a 12–25 model that acknowledges the non-linear nature of psychological development. Emerging adults are less likely to feel let down when transition care is sustained and structured. Transitions are seen as shared responsibilities, with adult services restructured to receive young people – unlike in the UK, where transition pathways are typically still embedded within child and adolescent mental health services (CAMHS), perpetuating disengagement at the point of greatest vulnerability.

Timely access to care is another defining fault-line. In Newcastle, New South Wales, embedding psychiatric nurses into emergency departments (EDs) allows young people to receive therapeutic assessments within 1 h of arrival. In the UK, young people in crisis often wait many hours or even days, often in overstimulating environments that can increase distress.^3^ Many leave before being seen, with each departure representing a missed opportunity for suicide prevention, while communicating the message that help, when sought, will not come.^4^

Perhaps the most philosophically charged difference lies in how risk is conceptualised. Australian services have begun to reject structured suicide risk assessment tools, recognising (in line with the UK’s National Institute for Health and Care Excellence (NICE) guidance^5^), that they neither predict nor prevent suicide.^6^ Instead, clinicians are encouraged to use collaborative formulations focused on context, strengths and goals. Risk is co-managed, not delegated; responsibility is shared, not outsourced to a tick-box form. These shifts challenge the bureaucratic reflexes that dominate UK culture, inviting a return to clinical judgement rooted in therapeutic relationships.

## Key innovations in Australian mental healthcare

### Early intervention: the headspace model

Australia’s national headspace centres serve as walk-in hubs for 12- to 25-year-olds, integrating mental, physical and sexual health with vocational support and peer-led care.^7^ These centres adopt a proactive, preventive ethos, offering low-threshold support before distress escalates. Emerging theories suggest that co-located, integrated services enhance engagement and satisfaction by building trust and reducing the need for young people to repeatedly tell their story.^8^ The headspace model’s ‘open door’ policy also represents the antithesis of the exclusion culture that dominates the UK’s National Health Service (NHS) mental healthcare provision.^9^ However, this model is not without limitations. Centres face challenges with staff retention and may lack the capacity to manage high-risk or complex presentations. Gaps also exist in accessing trauma and sexual assault services. Still, the underlying principle is powerful: early distress is not a diagnostic inconvenience, but a therapeutic opportunity.

### Assertive aftercare: the HOPE model

Structured aftercare is essential for emergency interventions to be meaningful. In contrast to the single-session assessments still common in UK liaison mental health services, Orygen’s HOPE (Hospital Outreach Post-suicidal Engagement) programme offers 12 weeks of assertive, community-based follow-up after a hospital-treated episode of self-harm.^10^ It is structured, systemic and flexible. A single coordinating clinician works alongside a multidisciplinary team, including peer workers, with contact tailored to individual need, whether through school visits, family meetings or outreach. Continuity is a central mechanism: sustained engagement with someone who listens without judgement. This model challenges the proceduralism of UK aftercare. It avoids pathologising distress or enforcing premature diagnosis, instead creating space for recovery to be co-constructed. Peer workers play a critical role in reducing power imbalances, modelling hope, and facilitating honesty and meaningful connection.

### Integration at the interface: School-Link, safeguards and PECCs

Australia has invested in integrated models that actively bridge sectoral silos. School-Link embeds mental health professionals within schools to train staff, support referrals and coordinate responses to mental health concerns with emergency services.^11^ Safeguards teams offer brief outreach interventions, often delivered at home or school, bridging crisis and community care.^12^ Psychiatric emergency care centres (PECCs) provide low-stimulation short-stay units for adults in crisis, co-located but distinct from EDs. These models reduce duplication, delays and fragmented narratives. Theories of integrated care suggest that successful navigation through mental health services depends not only on service availability but on coordination, communication and continuity.^13^ Warm handovers and shared narratives between services allow systems to adapt to young people, not the other way around.

## Telehealth, technology and equity

Australia is a global leader in the implementation of telehealth, particularly in youth mental healthcare. In rural areas, telepsychiatry assessments are routinely delivered within 24 h of ED presentation. Hybrid models, blending in-person and remote contact, offer flexibility and choice. When implemented well, telehealth enhances autonomy and reduces barriers to care, especially for young people who find clinical environments alienating. Its success, however, depends on infrastructure, training and integration. The UK is yet to consistently embed telehealth within CAMHS, despite its potential to reduce inequities and improve continuity.

## Workforce and systemic culture

A consistent feature across Australian services is the emphasis on relational care, staff well-being and reflective practice. Flexible working, trauma-informed training and multidisciplinary collaboration are standard. Flattened hierarchies foster psychological safety for both staff and patients. These cultural foundations are essential: even the most innovative models cannot succeed without a supported workforce. By contrast, UK services face rising staff burnout, often driven by risk-averse cultures, under-resourcing and pay erosion, which ultimately undermine relational care. Staff well-being is not a peripheral concern but central to therapeutic outcomes. When staff feel emotionally safe and professionally supported, they are more able to contain distress and build meaningful relationships with patients. Supporting our current and emerging workforce is essential to sustainable children’s mental health services, and the challenges currently faced in this sphere will only exacerbate if this remains unaddressed.

## Evaluating complex interventions: the evidence challenge

It is essential to acknowledge the challenges associated with evaluating complex mental health interventions. Australia’s willingness to innovate has not always been matched by robust evaluation. Many models were implemented without clear outcome frameworks, and there is little consensus on what constitutes success: reduced service use, improved functioning or trust in services? Without clarity, service metrics risk becoming proxies for success, masking the realities of unmet need, disengagement and even iatrogenic harm.

This is a familiar tension. Complex and relational interventions do not lend themselves easily to randomised controlled trials or narrow key performance indicators. Realist and complexity-informed methods are more nuanced alternatives, identifying mechanisms and contextual factors that drive impact.^14^ Medical Research Council guidance highlights that programme theories are useful to policymakers for evaluating complex interventions and transferable to different settings.^15^ If the UK is to adapt or adopt similar models, they must be coupled with evaluations that reflect the complexity of lived experience and the systems that serve it.

## Recommendations for the UK

Several lessons emerge for UK policymakers, commissioners, service providers and clinical leaders. Services must be designed around developmental need, not arbitrary age thresholds, recognising that young people’s needs do not change or disappear on their 18th birthday. Ineffective risk assessment tools should be retired and replaced with collaborative formulations that centre strengths and goals. Investment in early intervention hubs would provide low-threshold, integrated care for young people before distress escalates. Structured aftercare is essential to provide relational continuity and support beyond the immediate crisis. Outreach models must be consistently embedded to bridge EDs, schools, homes and social care to reduce fragmentation. Peer-led support should be implemented meaningfully, with proper training and remuneration. Staff well-being, supervision and reflective practice are not luxuries, but foundational to therapeutic care, and should therefore be prioritised.

Finally, evaluation frameworks must be designed to reflect the complexity of the real world and centre outcomes that matter to young people and their families. Above all, systems must stop asking distressed young people to prove their worthiness for help. Relationships, not thresholds or checklists, remain the surest and more humane route to safety and recovery.

## About the author

**Daniel Romeu** is an NIHR Doctoral Fellow at the University of Leeds, UK, and a higher trainee in child and adolescent psychiatry.
